# Development of joint movement patterns during gait in Japanese children aged 3–12 years

**DOI:** 10.1111/ped.70144

**Published:** 2025-07-21

**Authors:** Saori Miyagishima, Hiroki Mani, Yui Sato, Sota Hirosaki, Takaki Kurogi, Takashi Muchima, Takumi Aiko, Naoki Kozuka

**Affiliations:** ^1^ Division of Physical Therapy, Department of Rehabilitation, Faculty of Health Sciences Japan Healthcare University Sapporo‐shi Hokkaido Japan; ^2^ Division of Rehabilitation Sapporo Medical University Hospital Sapporo‐shi Hokkaido Japan; ^3^ Faculty of Welfare and Health Science Oita University Oita City Oita Japan; ^4^ Department of Health Promotion Science Tokyo Metropolitan University Hachioji‐shi Tokyo Japan; ^5^ Graduate School of Welfare and Health Sciences Oita University Oita City Oita Japan; ^6^ Department of Rehabilitation, Faculty of Health Science Hokkaido Chitose College of Rehabilitation Chitose‐shi Hokkaido Japan

**Keywords:** children, gait kinematics, joint movement, motion analysis, motor development

## Abstract

**Background:**

This study aimed to clarify the development of joint movement patterns during gait in Japanese children aged 3–12 years.

**Methods:**

The participants included 92 children (25 in the 3–4 to year‐old group, 28 in the 5–6 to year‐old group, 20 in the 7–8 to year‐old group, and 19 in the 9–12 to year‐old group) and 21 young adults (adult group). Kinematic data during gait were recorded using a three‐dimensional motion analysis system and four force plates.

**Results:**

The patterns of joint motion in the hip joint were similar across all the age groups. The maximum knee joint flexion angle during the swing phase was significantly greater in the 3–4‐year‐old and 5–6‐year‐old groups than in the adult group (*p* < 0.01). The minimum ankle joint angle during the loading response (LR) was significantly greater in the 3–4‐year‐old and 5–6‐year‐old groups than in the adult group (*p* < 0.01). Furthermore, the 7–8‐year‐old group did not exhibit the pattern observed in adults, where the ankle transitions from plantarflexion to dorsiflexion during the LR phase.

**Conclusions:**

Although the hip joint showed patterns similar to those of adults at all ages, knee joint motions were greater in 3–8‐year‐old children than in adults. In regard to ankle joint motion, the heel‐rocker function in the LR was suggested to mature after the age of 9 years.

## INTRODUCTION

Gait disorders are common in adults and children and are critical targets of physiotherapy interventions. Gait analysis, defined as the examination of structural and functional factors causing these disorders, is vital for treatment planning and outcome prediction.^1^ Observational gait analysis is simple and versatile but lacks accuracy. Advanced three‐dimensional motion analysis systems using infrared cameras provide precise joint kinematic data by tracking reflective markers, though they are limited by restricted measurement space, complex marker application, and challenges in children with developmental disabilities. Markerless motion capture, a recent innovation, enables natural gait assessment without markers, making it suitable for pediatric physical therapy.^2^ Gait is clinically divided into stance and swing phases, with further subdivisions to analyze deviations from adult‐like patterns, aiding in the evaluation of abnormal gait.^3^, ^4^ However, joint kinematic reference values for typical pediatric gait remain insufficiently established.

Sutherland et al. reported that the joint kinematics of typical gait in children follow the same pattern as adults by the age of 3.5–4 years,^5^ while others report that this pattern is only achieved by the age of 5 years,^6^ while others have reported the relevant age as 7 years and older.^7^, ^8^, ^9^ Ounpuu et al. investigated the joint movement patterns of children aged 5–16 years during gait and compared the results with those of adults, finding that the pattern is the same as that of adults by the age of 5 years; however, they have not revealed developmental changes because they did not compare between ages.^6^ Ganley and Powers, who reported that the gait pattern was the same as that of adults aged 7 years and older, also compared 7‐year‐old children with adults, and reported group differences in ankle joint motion; however, other age groups were not included, and developmental changes could not be revealed.^8^ In another study, Chester et al. examined 3–8‐year‐olds categorized in age groups by an interval of 2 years, with 9–13‐year‐olds in a single group, showing that 3–13‐year‐olds showed patterns similar to adults in the foot, knee, and hip joints, although there was no comparison with the adult group.^10^ In contrast, Ito et al. classified 6–12‐year‐old Japanese children into 6–8‐year‐old, 9–10‐year‐old, and 11–12‐year‐old groups and reported that joint movements of the hip and knee joints gradually decreased after the age of 9–10 years, and that there were no significant differences in the foot joints among the age groups.^11^ Interestingly, they further mentioned the possibility that children in our country and those in other countries would show different trends in joint kinematics.^11^ As described above, there is no consistent view on the joint kinematics of typical gait in children. However, there have been very few reports on joint motion patterns during gait in children under 6 years of age, and the collection of data unique to Japan is inadequate.

The purpose of this study was to clarify the developmental changes in joint kinematics during gait in Japanese children aged 3–12 years. First, we visualized the joint kinematics during walking for each age group, and then verified the developmental changes. Subsequently, we statistically verified the phases exhibiting these characteristics. We hypothesized that (1) knee and hip joint motions in children would show the same patterns as those in adults, but that joint angles would decrease in children compared to adults, and (2) ankle joint motions would show the same patterns and joint angles as those in adults. To evaluate normal gait, it is essential to clarify the kinematic characteristics of gait development in children. The findings of this study elucidate the kinematic characteristics of pediatric gait in Japan and are expected to serve as fundamental data for clinical gait analysis and video motion analysis using artificial intelligence (AI) technology.

## METHODS

### Subjects

Participants were recruited from Sapporo Medical University between April 1, 2016 and December 1, 2022. Recruitment was through advertisements within the university and direct referrals from research staff.

A total of 113 participants, including 92 typically developing children aged 3–12 years and 21 healthy adults, were included in this study. The inclusion criteria required that children were aged 3–12 years, had achieved typical developmental milestones as confirmed through a parent‐completed questionnaire, and had parental consent. For adult participants, the inclusion criteria required that they were aged 21 years or older, in good health, and had provided individual consent. The exclusion criteria, applicable to all participants, included the presence of central nervous system disease, orthopedic disease, pain, or other conditions that could affect the ability to maintain a one‐legged standing position. To ensure eligibility, the parents of child participants completed a questionnaire to confirm the absence of developmental delays, to provide information on the timing of acquisition of major gross motor skills, and to confirm that the children showed no obvious delays in gross motor development. For adults, self‐reported medical history and physical condition were reviewed to confirm they met the health requirements for participation.

Sample size was calculated based on the “ankle joint angle during loading response (LR)” in our pilot study. The mean and standard deviation (SD) of five persons in each group from this preliminary experiment were used to determine the desired sample size. Based on maximum pairwise comparisons, the sample size needed for this study was approximately 18 persons in each group. The calculation was performed with a significance level (α) of 0.05, a statistical power (1‐β) of 0.8, and a two‐tailed test.

In accordance with the Declaration of Helsinki and the ethical guidelines for medical research involving human subjects issued by the Ministry of Health, Labor and Welfare and the Ministry of Education, Culture, Sports, Science, and Technology, this study was conducted after the subjects and their guardians were fully informed of the purpose of the research through a research explanation document, while consent was obtained from the research cooperation consent form. This study was approved by the Sapporo Medical University Ethics Committee and Oita University Faculty of Welfare and Health Sciences Ethics Committee (Sapporo Medical University approval number: 28‐2‐52, Oita University approval number: F220002).

### Measurement methods

#### Experimental procedure and subject

Before starting the measurement, the subjects changed into lightweight experimental clothing, and their height, weight, and lower limb length were measured. The lower limb length was defined as the distance from the greater trochanter to the floor.

A total of 27 infrared reflective markers, each with a diameter of 9.5 mm, were placed on the subject's skin over the body bone landmarks. A full‐body model with 27 reflective markers was used in this study as it was shared with other analyses conducted simultaneously as part of a broader research project. The markers were placed so that the anthropometric data of Jensen^12^ could be used to calculate the somatoskeletal characteristics (top of the head, foramen auricularis, seventh cervical vertebra, sternum, both sides of the acromion, lateral upper carpal bones on both sides, central hand joints on both sides, both sides of the third middle hand head, upper anterior bowel spine on both sides, upper posterior bowel spine on both sides, bilateral big spinners, knee joint cleft (lateral) on both sides, external fruit on both sides, both sides of the heel bone, and both second midfoot bones).

**FIGURE 1:**
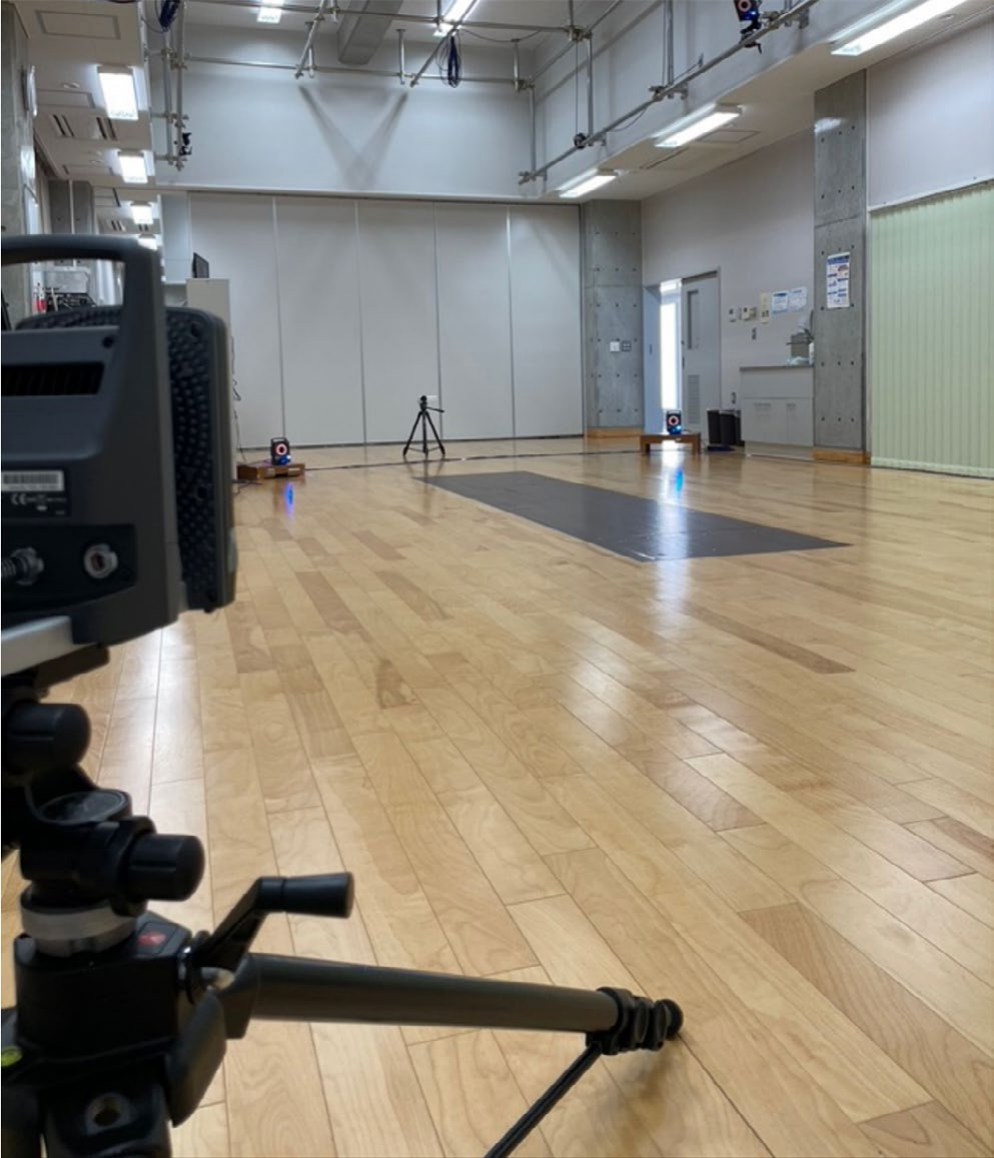
Experimental environment.

Measurements were taken in a room equipped with a motion analysis system at Sapporo Medical University and Oita University. The subjects were instructed to walk barefoot along a 6 m gait path at their comfortable speed, during which kinematic data were recorded using a 10‐camera VICON Nexus three‐dimensional motion analysis system (camera: MX‐T‐10S, Nexus2.11, USA) and four force plates (9281E, Kistler) during gait (Figure 1). The sample frequencies of the 3D motion analysis system and the force plate were 100 and 1000 Hz, respectively, and the signals were recorded synchronously. To negate the effects of acceleration and deceleration at the start and stop of the gait, the position of the floor reaction force meter was set at 2–4 m in the middle. Starting was always performed in the same position. After several practice sessions, the test was conducted five times in total. To ensure reliability, the results of these trials were averaged. Additionally, to minimize the effects of fatigue, participants were allowed to take breaks of a few minutes at their own discretion between trials.

#### Data analysis

All signals were processed off‐line using MATLAB2022a (MathWorks, Natick, MA, USA). Both the three‐dimensional coordinate data and floor reaction force signals were processed using a fourth‐order Butterworth low‐pass filter (cutoff frequency, 20 Hz). The motions of the hip, knee, and ankle joints in the sagittal plane were calculated using the coordinate data of the reflex markers. The hip joint was defined as the angle between the line connecting the acromion and the greater trochanter and the line connecting the greater trochanter and the knee joint cleft; the knee joint was defined as the angle between the line connecting the greater trochanter and the knee joint cleft and the line connecting the knee joint cleft and the lateral malleolus; and the ankle joint was defined as the angle between the line connecting the knee joint cleft and the lateral malleolus and the line connecting the calcaneal prominence and the second metatarsal head. The hip and knee joints were calculated to be in flexion and the ankle joint was calculated to be in dorsiflexion when values were positive. The first heel contact (T_0_) was defined as the first time the vertical force of the floor reaction force exceeded 5 N, while the next heel contact (T_1_) was defined as the time when the vertical force of the ipsilateral lower limb exceeded 5 N after the free leg. In our previous study, we reported that the gait pattern, time factor, and symmetry were equivalent to those of adults by 7 years of age, while variability and stability did not reach the adult level, even at 10 years of age.^13^ Also, we confirmed that gait variables normalized by body size were not significantly different from those of adults in step time, support leg time, and swing leg time from approximately 3 to 4 years of age; we assumed 60% for the stance phase and 40% for the swing leg phase.^13^ Therefore, we defined the stance phase (0–60%) as 0–12% LR, 12–40% mid‐stride, and 40–60% stance cycle.

We calculated the maximum and minimum values of each joint angle during the stance and swing phases, respectively, the ankle joint angle at heel contact (at T_0_), and the minimum ankle joint angle during LR (0–12% stance phase).

### Statistical analysis

The Shapiro–Wilk test was first used to check the normality of the distribution of each variable. If the variables were normally distributed, one‐way analysis of variance (ANOVA) was applied, while the Kruskal–Wallis test was used for non‐normally distributed variables. When significant differences were found, multiple comparisons were made using the Tukey–Kramer method for variables subjected to one‐way ANOVA and the Steel‐Dwass test for variables subjected to the Kruskal‐Wallis test as post‐hoc tests. All statistical analyses were performed using the statistical software R (version 4.2.1). The significance level was set at 5%.

## RESULTS

**FIGURE 2:**
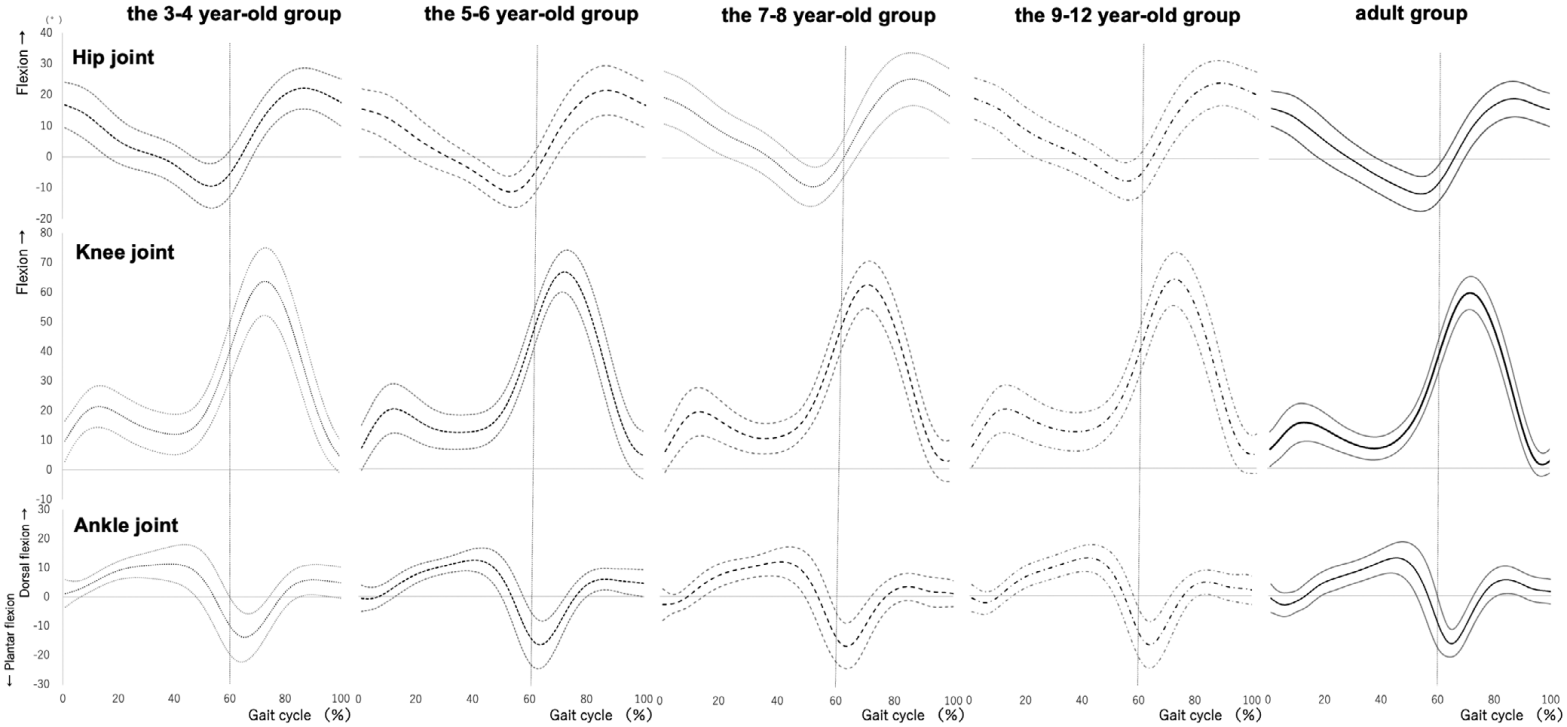
The joint motion patterns during gait for each age group. The joint motion of each age group during one gait cycle is shown. The horizontal axis shows the gait cycle as a percentage. The vertical axis shows the joint angle (°). The dark line in the center represents the mean value, and the light lines at the top and bottom are ± standard deviation. The dotted line in front of the 60th percentile indicates the stance phase, and the dotted line after the 60th percentile indicates the swing phase.

**FIGURE 3:**
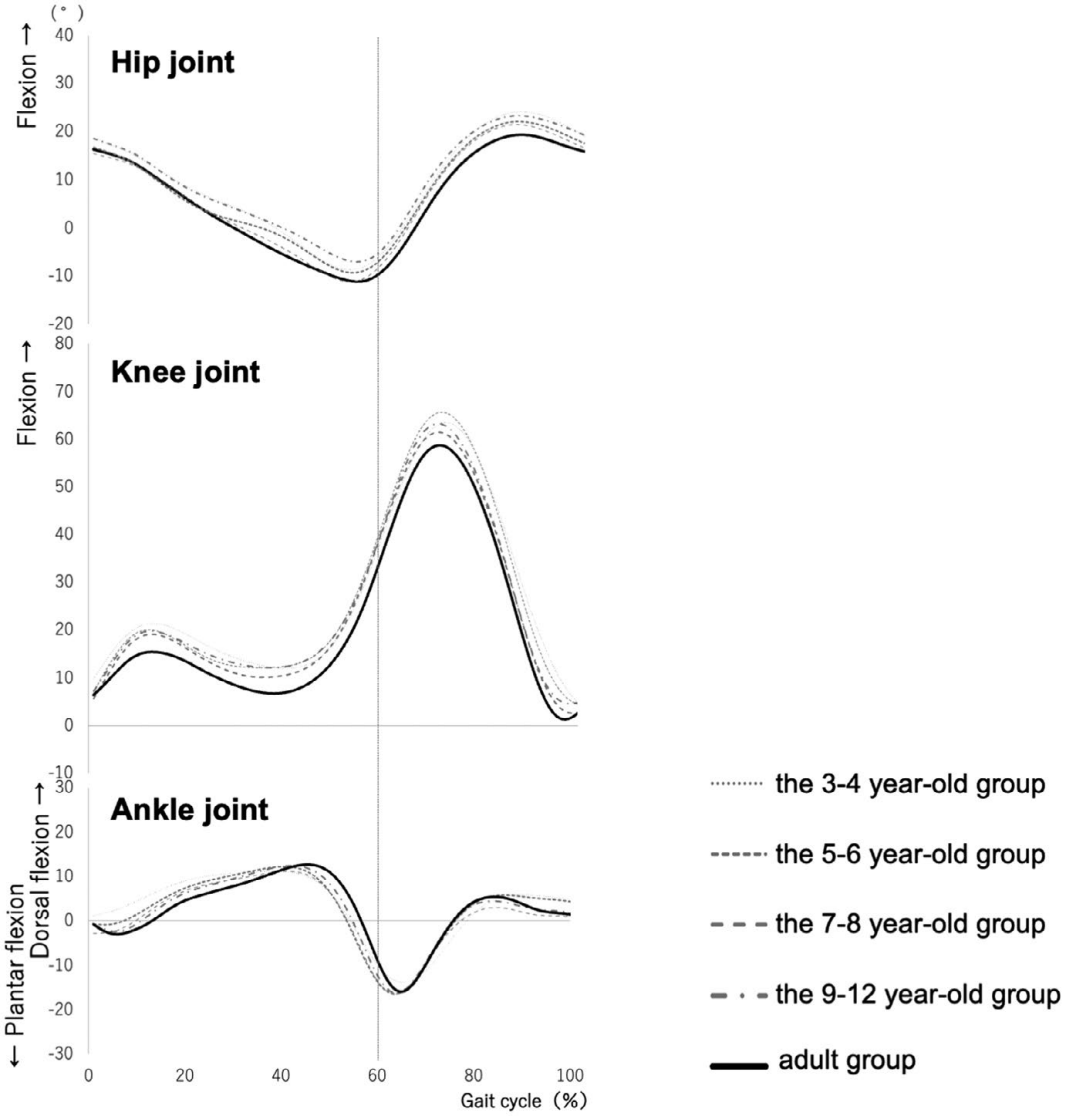
Mean values of each joint motion for each age group. The mean values of each joint motion during one gait cycle are shown in the same graph for all groups. The horizontal axis shows the gait cycle as a percentage. The vertical axis shows the joint angle (°). The dotted line in front of the 60% part represents the stance phase, and the dotted line after the 60% part represents the swing phase.

The children were assigned to the following groups based on age:3–4 to year‐old (*n* = 25),5–6 to year‐old (*n* = 28),7–8 to year‐old (*n* = 20), and 9–12 (*n* = 19). The physical characteristics of each group are presented in Table 1. Figure 2 shows the joint motion patterns during one gait cycle for each age group, as the mean and standard deviation of all the subjects in each group. Figure 3 shows the average waveforms of joint motion for all groups overlaid on the graph. Table 2 shows the maximum and minimum values of each joint angle, the ankle joint angle at heel contact, and the ankle joint angle during LR for the stance and swing phases.

**TABLE 1:** Basic information of the study subjects.

	3–4 years group (*n* = 25)	5–6 years group (*n* = 28)	7–8 years group (*n* = 20)	9–12 years group (*n* = 19)	Adult group (*n* = 21)
Boys (male)	14	13	11	12	11
Girls (female)	11	15	9	7	10
Age (years)	4.1 ± 0.6	6.0 ± 0.5	7.8 ± 0.5	10.2 ± 1.0	22.3 ± 2.3
Height (cm)	101.6 ± 8.0	113.0 ± 5.6	123.7 ± 6.5	136.5 ± 9.4	168.0 ± 6.8
Weight (kg)	16.3 ± 2.7	20.1 ± 3.3	23.6 ± 2.8	29.0 ± 5.2	60.9 ± 10.2
Leg length (cm)	45.4 ± 4.0	53.2 ± 3.7	60.9 ± 4.2	67.6 ± 5.4	83.4 ± 4.4

**TABLE 2:** Minimum and maximum values of each joint angle during gait, ankle joint angle at the heel ground contact, and the mean of minimum values of the LR ankle joint angle (standard deviation).

	3–4 years group *n* = 25 (mean)	5–6 years group *n* = 28 (mean)	7–8 years group *n* = 20 (mean)	9–12 years group *n* = 19 (mean)	Adult group *n* = 21 (mean)	*p* value (Kruskal‐Wallis test = K‐W)	Effect size (*η* ^2^) *only one‐way ANOVA	Group differences Tukey–Kramer test Steel‐Dwass test
Stance phase								
Hip joint								
Maximum	18.40	16.88	15.98	18.46	16.65	0.69	0.02	
	(8.08)	(7.41)	(6.36)	(6.35)	(5.58)			
Minimum	−9.05	−9.72	−10.60	−7.40	−11.73	0.26	0.05	
	(5.95)	(7.03)	(5.95)	(5.94)	(5.46)			
Knee joint								
Maximum	39.85	41.59	38.99	40.78	36.02	0.07	0.08	
	(8.50)	(6.27)	(9.97)	(7.80)	(5.23)			
Minimum	8.07	6.74	4.52	6.66	4.17	0.34	0.05	
	(5.68)	(7.62)	(7.16)	(7.05)	(4.13)			
Ankle joint								
Maximum	13.36	13.06	14.02	12.91	13.13	K‐W 0.97		
	(4.45)	(3.42)	(7.32)	(4.31)	(5.06)			
Minimum	−10.64	−13.94	−12.61	−12.59	−10.75	0.50	0.03	
	(8.40)	(8.64)	(7.83)	(7.57)	(5.90)			
Ankle angle at heel contact	1.16	−0.71	−2.68	−0.79	−0.79	0.12	0.06	
	(4.71)	(4.40)	(5.26)	(4.47)	(4.76)			
The minimum ankle Angle during LR	0.51	−0.80	−4.17	−2.84	−4.43	K‐W >0.01		3–4 years group vs. adult group *p* < 0.01 5–6 years group vs. adult group *p* < 0.01
	(3.79)	(2.54)	(4.04)	(3.70)	(3.54)			
Swing phase								
Hip joint								
Maximum	24.54	22.58	21.58	23.70	19.77	0.16	0.06	
	(7.95)	(6.75)	(8.19)	(6.74)	(4.59)			
Minimum	−3.24	−4.28	−4.73	−2.50	−7.18	0.81	0.01	
	(5.54)	(7.33)	(6.70)	(6.69)	(5.31)			
Knee joint								
Maximum	64.50	66.10	59.82	63.98	58.99	K‐W >0.01		3–4 years group vs. adult group *p* < 0.01 5–6 years group vs. adult group *p* < 0.01
	(7.95)	(6.75)	(8.19)	(6.74)	(5.56)			
Minimum	8.07	6.74	4.52	6.66	3.83	0.15	0.06	
	(6.13)	(7.22)	(7.76)	(5.65)	(3.52)			
Ankle joint								
Maximum	7.10	6.68	7.43	5.03	5.58	K‐W 0.48		
	(5.00)	(3.53)	(12.88)	(3.93)	(4.43)			
Minimum	−14.95	−16.92	−16.63	−17.15	−16.98	0.81	0.01	
	(7.42)	(8.02)	(7.78)	(7.62)	(4.21)			

The pattern of hip joint motion was similar in all age groups (Figure 2, top row; Figure 3, top row), and there was no significant main effect of the hip joint angle between the groups. In regard to the knee joint, the pattern of joint motion was similar for all age groups (middle row of Figure 2 and middle row of Figure 3), but we found a significant main effect between groups for the maximum knee joint angle (flexion angle) during the second knee action of the swing phase (*χ* ^2^ = 16.68, *df* = 4, *p <* 0.01). The maximum knee joint flexion angle was significantly greater in the 3–4‐year‐old and 5–6‐year‐old groups than in the adult group (*p* < 0.01) (Table 2). Although no significant differences were found, the maximum knee joint angle (flexion angle) of the first knee action during the stance phase tended to be larger in all the child groups than in the adult group (*p* = 0.07).

The ankle joint motion patterns showed different characteristics in LR for each age group. In the adult group and the 9–12‐year‐old groups, the ankle joint was in an intermediate position of plantar‐dorsiflexion (approximately 0 degrees) during heel contact (HC) (gait cycle 0%), followed by plantar flexion once, and then dorsiflexion during LR (Figure 2, lower panel). In contrast, the 3–4‐year‐old, 5–6‐year‐old, and 7–8‐year‐old groups did not show the same pattern as the adult group (Figure 3, lower row). These groups showed ankle dorsiflexion was gradually increased during LR. There was a significant main effect of ankle joint angle at LR (*χ* ^2^ = 26.52, *df* = 4, *p* < 0.01) (Table 2). The 3–4‐year‐old and 5–6‐year‐old groups had significantly larger ankle joint angles at LR than the adult group (p < 0.01) (Table 2).

## DISCUSSION

This study aimed to clarify the developmental changes in joint kinematics during gait in Japanese children aged 3–12 years. The children were classified into four groups (3–4, 5–6, 7–8, and 9–12 years‐old), with an adult control group. Joint kinematics during gait were visualized and statistically examined for each age group.

The pattern of motion in the sagittal plane of adults was similar to the pattern of normal joint motion shown by Perry et al.,^4^ the most commonly cited study, while the results of the joint motion in adults support those of previous studies. The results of this study further revealed that, in partial contrast to the aforementioned Hypothesis (1), the hip joint showed patterns similar to those of adults at all ages, but the maximum knee joint angle (flexion angle) was greater in the 3–4‐year‐old and 5–6‐year‐old groups than in the adult group during the second knee action in the swing leg phase. In regard to hypothesis (2), the ankle joint motion patterns in the LR group showed the same patterns as those in adults after the age of 9 years and were different for each age group. The 3–4‐year‐old and 5–6‐year‐old groups had significantly larger ankle joint angles at the LR than the adult group, which did not agree with Hypothesis (2).

The results of the present study differ from those reported by Chester et al.^10^ who found the same pattern of hip and knee joint motions in 3–13‐year‐old children. In contrast, Ito et al. showed that the hip and knee joint motions in the sagittal plane gradually decreased after the age of 11 years,^11^ a trend that was partially similar to the results of the present study, although we used different age categories. Furthermore, Ito et al. showed that the maximum knee joint flexion angle during the swing phase decreases in children aged 11–12 years, and that the range of motion of the knee joint during the gait cycle also decreases. They claimed that this result was influenced by the shorter stride length of the children.^11^ In our previous study,^13^ the stride length normalized by the lower limb length was significantly shorter in the 3–4‐year‐old and 5–6‐year‐old groups than in the adult group, suggesting that the maximum knee joint angle (flexion angle) was increased in the 3–6‐year‐old children in this study compared with that in adults owing to differences in stride length.

The ankle joint angles during LR were significantly larger in the 3–4‐year‐old and 5–6‐year‐old groups than in the adult group. This result indicates that younger children showed higher ankle dorsiflexion, and the ankle joint plantar flexion movement associated with heel contact during LR is small, meaning that the heel rocker function was not yet developed. There was no significant difference between the 7–8‐year‐old group and the adult group. However, the 7–8‐year‐old group showed a large individual variation at the HC time point (0% gait cycle), and the curve pattern differed from that of the adult group (Figure 3, lower row). These results differ from those of previous studies.^10^, ^11^ In one prior study, Chester et al. reported that ankle joint moments were significantly smaller in a 3–4‐year‐old group than in other age groups, whereas joint motions were the same in each age group.^10^ Ito et al. also reported that the ankle joints did not differ significantly between age groups.^11^ These studies were comparisons between age groups of children, but not between age groups of adults. Since ankle joint motion changes gradually with age (Figure 3, lower row), it is possible that developmental changes in the ankle joint could not be detected by comparison between age groups and that significant differences were found by comparison with the adult group, as in the present study. In addition, the present study focused not only on the maximum angle during one gait cycle but also on each phase of gait from HC to LR, which may also have contributed to the identification of developmental changes. Furthermore, the results of a study by Ito et al. showed that the characteristics of moments and gait in Japanese children differ from those of children in other countries,^11^ suggesting that the characteristics of the present study may reflect gait development in Japanese children. The results of this study suggest that the heel rocker function in the LR develops after the age of 9 years.

This study has several limitations. First, the accuracy of the joint angles remains an issue. The number of markers in this study was minimized for younger children, while the axes connecting the markers were used as the basic and measurement axes. Therefore, the axes of measurement of the joint angles are different from those used in clinical evaluation. However, because the joint motions of adults were similar to those in previous studies,^3^ while the method was similar to the segment construction of the markerless motion capture skeletal recognition technique, the results of this study can be used as a reference value when using the markerless motion capture system. Second, the validity of the county‐level classification method was not examined. Although previous studies have used various methods of group division and have shown some trends, it may be necessary to increase the number of subjects, classify them by age, and re‐examine the results to clarify the developmental changes with increasing age. Third, while pelvic tilt has a significant impact on the movements of the hip, knee, and ankle joints, the marker set used in this study was designed to minimize the number of markers for feasibility with younger children. Although pelvic tilt could be estimated as the angle formed between the line connecting the anterior superior iliac spine (ASIS) and posterior superior iliac spine (PSIS) and the horizontal plane, this approach lacks sufficient validity and reliability with the current marker set. As such, calculating pelvic tilt was deemed impractical in this study. However, we recognize the importance of analyzing pelvic tilt for understanding gait development, and this remains an area for future research.

In order to evaluate normal gait, basic data as a reference standard is necessary. This study is the first report in Japan that clarified joint motion patterns during gait in children, including younger children. It is expected to serve as basic data for gait analysis in clinical settings and for gait analysis using video technology such as marker‐less motion capture utilizing AI, which is expected to make further progress in the future.

## CONCLUSIONS

Overall, this study examined the development of joint movement patterns during gait in 113 subjects, including 92 typically developing children aged 3–12 years and 21 healthy adults. The hip and knee joints showed patterns similar to those observed in adults of all ages. However, sagittal plane motion of the knee joint showed a greater flexion angle in children than in adults. Further, the ankle joint motion showed a pattern similar to that of adults after the age of 9 years. In particular, the heel rocker function in the LR was suggested to develop after the ages of 9–12 years. To assess typical gait, it is important to characterize the kinematics of pediatric gait according to its development. In addition, basic data are needed to evaluate typical developments. This study is the first in Japan to clarify joint movement patterns during gait, including during infancy, and is expected to be utilized in clinical practice and future research to help identify the causes of gait disorders and formulate treatment strategies.

## AUTHOR CONTRIBUTIONS

S.M. and M.H. designed the study; S.M., M.H., Y.S., S.H., T.K., M.T., and T.A. performed experiments, collected, and analyzed data. S.M. and M.H. wrote the manuscript; N.K. gave technical support and conceptual advice. All authors read and approved the final manuscript.

## FUNDING INFORMATION

This research was supported by JSPS Grants‐in‐Aid for Scientific Research JP19K19901, JP22K11289, JP24K02423, and the President's Strategic Fund of Oita University.

## CONFLICT OF INTEREST STATEMENT

The authors declare no conflict of interest.
